# Optimizing the surgical management of MRI‐negative epilepsy in the neuromodulation era

**DOI:** 10.1002/epi4.12578

**Published:** 2022-02-01

**Authors:** Hari McGrath, Mauricio Mandel, Mani Ratnesh S. Sandhu, Layton Lamsam, Nana Adenu‐Mensah, Pue Farooque, Dennis D. Spencer, Eyiyemisi C. Damisah

**Affiliations:** ^1^ Department of Neurosurgery Yale School of Medicine Yale University New Haven Connecticut USA; ^2^ Department of Neurology Yale School of Medicine Yale University New Haven Connecticut USA; ^3^ Department of Neuroscience Yale School of Medicine Yale University New Haven Connecticut USA

**Keywords:** epilepsy surgery, Intracranial EEG, intractable epilepsy, MRI negative epilepsy

## Abstract

**Objective:**

To evaluate the role of intracranial electroencephalography monitoring in diagnosing and directing the appropriate therapy for MRI‐negative epilepsy and to present the surgical outcomes of patients following treatment.

**Methods:**

Retrospective chart review between 2015‐2021 at a single institution identified 48 patients with no lesion on MRI, who received surgical intervention for their epilepsy. The outcomes assessed were the surgical treatment performed and the International League Against Epilepsy seizure outcomes at 1 year of follow‐up.

**Results:**

Eleven patients underwent surgery without invasive monitoring, including vagus nerve stimulation (10%), deep brain stimulation (8%), laser interstitial thermal therapy (2%), and callosotomy (2%). The remaining 37 patients received invasive monitoring followed by resection (35%), responsive neurostimulation (21%), and deep brain stimulation (15%) or no treatment (6%). At 1 year postoperatively, 39% were Class 1‐2, 36% were Class 3‐4 and 24% were Class 5. More patients with Class 1‐2 or 3‐4 outcomes underwent invasive monitoring (100% and 83% respectively) compared with those with poor outcomes (25%, *P* < .001). Patients with Class 1‐2 outcomes more commonly underwent resection or responsive neurostimulation: 69% and 31%, respectively (*P* < .001).

**Significance:**

The optimal management of MRI‐negative focal epilepsy may involve invasive monitoring followed by resection or responsive neurostimulation in most cases, as these treatments were associated with the best seizure outcomes in our cohort. Unless multifocal onset is clear from the noninvasive evaluation, invasive monitoring is preferred before pursuing deep brain stimulation or vagal nerve stimulation directly.

**Key points**

- Forty‐eight patients with medically intractable MRI‐negative epilepsy received invasive monitoring and/or definitive surgical treatment over 6 years.
- The treatments conferring the best outcomes (ILAE 1‐2) were resection or RNS which required invasive monitoring in all cases.
- Invasive monitoring is recommended in most cases followed by focal treatment, if indicated, consisting of resection or RNS.
- Noninvasive findings strongly indicating a multifocal onset, namely a nonlocalizing ictal scalp EEG, may support direct VNS or DBS therapy.

## 1 INTRODUCTION

The success of epilepsy surgery is determined by the treatment team's ability to localize, resect, disconnect, or modulate the epileptogenic region or network.^1^ About 20% to 40% of adult intractable epilepsy patients have no lesion on MRI, which can portend a poor surgical outcome due to the challenge of seizure onset localization.^2^, ^3^, ^4^ There are a number of preoperative predictors of seizure outcome following resective surgery for MRI‐negative epilepsy. These include the localization of 18‐fluorodeoxyglucose positron emission tomography (FDG‐PET) and ictal single‐photon positron emission tomography (SPECT) scans, as well as the concordance of multiple diagnostic modalities to a single brain region.^5^, ^6^ While the literature recognizes the importance of precise seizure localization for targeting treatment in MRI‐negative cases, the reports which have critically evaluated the use of intracranial electroencephalography (icEEG) monitoring are limited.^7^, ^8^

Previous studies have shown that resective surgery in MRI‐negative epilepsy achieves Engel Class 1 in 37%‐60% of patients.^9^, ^10^, ^11^ Since the introduction of deep brain stimulation (DBS), which can decrease seizure frequency in MRI‐negative epilepsy and does not require localization of an epileptogenic focus, the role of icEEG monitoring for seizure onset localization in MRI‐negative epilepsy cases is less clear.^12^ However, optimal seizure control in focal epilepsy may require resection and/or responsive neurostimulation (RNS), both of which are reliant on precise ictal localization. Our study assesses the use of diagnostic evaluations in determining the optimal surgical treatment in patients with MRI‐negative, refractory, focal epilepsy as well as those patients' outcomes.

## 2 MATERIALS AND METHODS

We retrospectively reviewed the clinical records of all patients with medically intractable focal epilepsy who presented to the Yale Epilepsy Surgery Program surgical conference between 2015 and 2021. Of the 477 patients, 175 (36.7%) had no lesion on preoperative 3 Tesla MRI. Forty‐eight consecutive patients who underwent icEEG monitoring or direct surgical treatment for epilepsy were included in this study. The majority of the remaining patients were maintained on medical therapy with a change to their regimen by the clinical team. Some patients chose not to undergo surgery or had surgery at another center. The cohort consisted of 23 men and 25 women of ages 15‐70 years (mean, 33 ± 13). The age at onset of epilepsy ranged from 1 month to 47 years (mean, 15 ± 12.6), and the duration of epilepsy prior to surgical evaluation ranged from 2 to 49 years (mean, 18 ± 9.69). Duration of follow‐up ranged between 3 and 74 months (mean, 32 ± 23.6); patients with less than 12 months of follow‐up were excluded from outcome analysis.

### 2.1 Preoperative evaluation

All patients underwent a comprehensive preoperative work‐up consisting of history, physical, and neurological examination, imaging, scalp electroencephalography, and neuropsychological testing. A 3 Tesla brain MRI was performed in all patients and was reviewed by a neuroradiologist for structural abnormalities. All but three patients underwent 18‐fluorodeoxyglucose positron emission tomography (PET), and ictal single‐photon emission tomography (SPECT) was performed in 20/48 patients (41.7%). For language lateralization, functional MRI (fMRI) was performed in 44 patients (91.7%). When this was inconclusive, an intracarotid amobarbital test was performed (9 patients, 18.8%). A standard battery of neuropsychological testing was performed in all patients. Concordance of evaluations was defined as lateralization to the same hemisphere and localization to the same lobe. The decision to offer patients icEEG monitoring was made on a case by case basis. In general, icEEG monitoring was offered when noninvasive studies could lateralize but not localize seizure onset, when detailed extra operative language mapping was required, and in some cases, when there was no lateralization based on the noninvasive studies. The final decision was made by consensus at a multidisciplinary epilepsy surgery conference.

### 2.2 Surgical intervention

Intracranial EEG monitoring consisted either of grid, strip, and depth electrodes (“combined monitoring”) or of depth electrodes only – the decision was made on a case by case basis. Combined monitoring was generally used when the preoperative evaluation was able to lateralize the seizure onset, although some early cases relied on bilateral craniotomies with depths, strips, and grids. Depth electrode‐only monitoring was typically used when the preoperative data was non‐lateralizing. The decision to pursue resection or neuromodulation was made at multidisciplinary conference based on the findings of the noninvasive and invasive investigations.

### 2.3 Follow‐up

ILAE seizure outcomes were assessed for patients with at least 1‐year follow‐up after definitive surgical treatment and were split into Excellent (ILAE 1‐2), Good (ILAE 3‐4) and Poor (ILAE 5).^13^ Patients who underwent corpus callosotomy were excluded from outcome analysis.

### 2.4 Statistical analysis

Three separate analyses were performed: preoperative predictors of performing icEEG monitoring, predictors that led to resection vs non‐resective treatment (including DBS, corpus callosotomy, LITT, RNS, and VNS), and outcomes analysis. Continuous variables were subjected to two‐tailed *t*‐tests or nonparametric Mann‐Whitney *U* Test, and categorical variables were subjected to χ^2^ or Fisher's exact tests. Univariate analysis was performed on significant variables using binomial logistic regression. Significance was defined as *P* < .05. Analysis was performed in R (version 4.0.3) and GraphPad Prism (version 9).

## 3 RESULTS

### 3.1 Diagnostics

**FIGURE 1:**
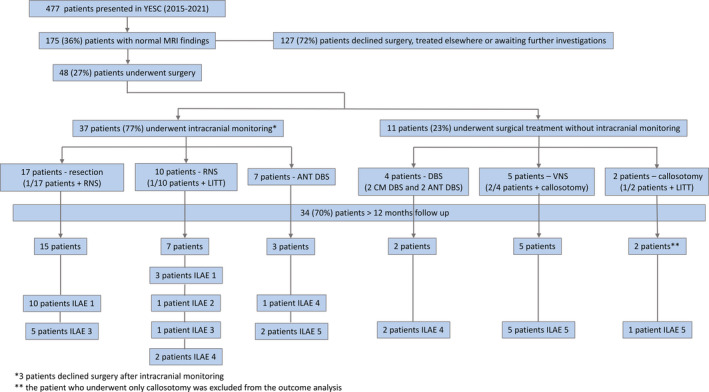
A decision‐tree outlining the diagnostic and treatment algorithms

Scalp EEG and PET most commonly localized the epileptogenic region to the temporal lobe in 22/48 (45.8%) and 27/44 (61.4%) patients, respectively (Table S1). Neuropsychological evaluation was most commonly non‐lateralizing (23/44, 52.3%) or indicated a left hemisphere deficit (14/44, 31.8%). Of the 48 patients, 37 (77.1%) underwent icEEG monitoring for localization of the epileptogenic region while 11 patients (22.9%) underwent direct surgical treatment without icEEG monitoring (Figure 1). One patient who received icEEG monitoring did not complete the evaluation. The mesial temporal lobe was the most common area of the icEEG ictal onset (10/36, 27.8%), followed by the temporal neocortex (6/36, 16.7%), frontal lobe (4/36, 11.1%), and multifocal onset (7/36, 19.4%).

### 3.2 Invasive monitoring

A localizing ictal scalp EEG onset area and a localizing semiology were associated with performing icEEG monitoring (*P* < .001 and *P* = .01 respectively). The most common type of icEEG monitoring involved a combination of grid, strip, and depth electrodes (24/37, 64.9%). Thirteen patients (35.1%) had depth alone monitoring due to non‐lateralizing preoperative evaluations. Of those, four went to resection, four to DBS, and three to RNS; the remainder did not have definitive surgical treatment. Other noninvasive preoperative evaluations including neuropsychology, interictal scalp EEG, PET and SPECT had no association with icEEG monitoring (Table 1). Intracranial EEG monitoring was associated with female patients (*P* = .016), patients with fewer seizures per month (28.1 vs 80.2, *P* = .023) and patients who had been trialed on fewer AEDs prior to surgery (7.41 vs 11.4, *P* = .034). Two patients had complications from icEEG monitoring: one developed a lower extremity deep venous thrombosis and the other had intraventricular hemorrhage with subsequent obstructive hydrocephalus. Both patients had complete resolution of their symptoms at 1‐month follow‐up. Three patients had no definitive treatment following icEEG monitoring, which included the two patients who suffered complications following icEEG monitoring and another patient who declined further surgery.

**TABLE 1:** A comparison of preoperative characteristics of the cohort that received icEEG monitoring (icEEG) and the cohort that did not receive icEEG monitoring (No icEEG)

Variable	icEEG	No icEEG	*P* value
Sex (female)*	23 (62.2%)	2 (18.2%)	.016
Number of seizures per month*	28.1 (± 42.8)	80.2 (± 110)	.023
Number of AEDs trialed (total)*	7.41 (± 3.36)	11.4 (± 5.41)	.034
Semiology lateralization (lateralized)	20 (54.1%)	3 (27.3%)	.173
Semiology localization (localized)*	31 (83.8%)	5 (45.5%)	.01
Neuropsychology lateralization (lateralized)	19 (52.8%)	2 (25%)	.245
Neuropsychology lateralization (language‐dom)	12 (70.6%)	2 (100%)	1
Interictal EEG lateralization (right)	23 (62.2%)	4 (36.4%)	.174
Interictal EEG localization (localized)	31 (83.8%)	7 (63.6%)	.206
Ictal EEG lateralization (right)	14 (37.8%)	1 (9.1%)	.136
Ictal scalp EEG localization (localized)*	34 (94.4%)	5 (45.5%)	<.001
PET lateralization (right)	15 (40.5%)	3 (33.3%)	1
PET localization (localized)	26 (72.2%)	5 (55.6%)	.428
SPECT lateralization (right)	4 (26.7%)	0 (0.0%)	.530
SPECT localization (localized)	9 (69.2%)	3 (75.0%)	1

Note

*P ≤ .05.

### 3.3 Surgical treatment

**FIGURE 2:**
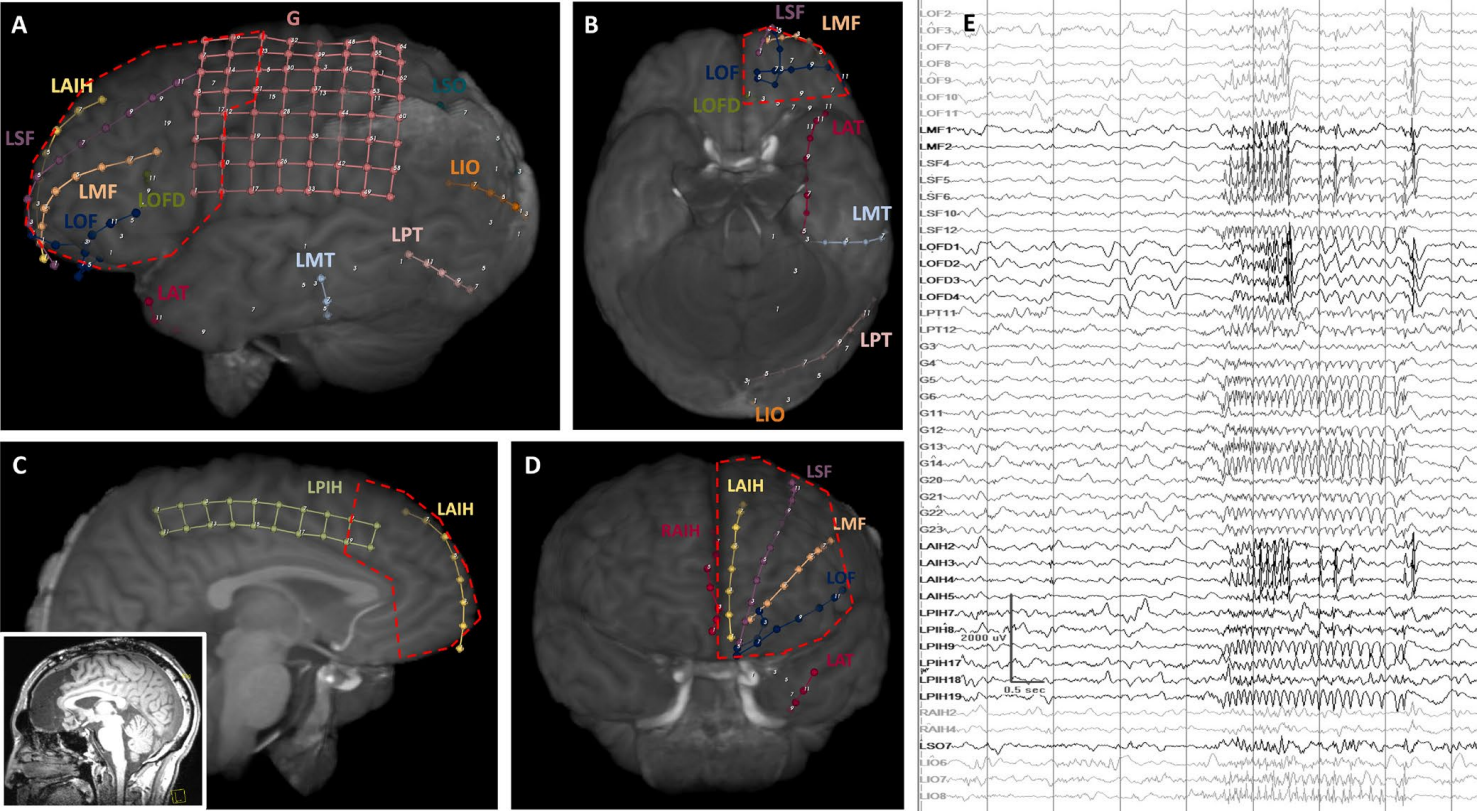
An illustrative case of a left‐handed, right hemisphere dominant male with a history of focal aware and focal to bilateral tonic clonic seizures who underwent resective treatment. The combined icEEG monitoring consisted of a fronto‐parietal 8 × 8 grid over the motor and sensory cortices and strips over the adjacent cortex. The onset was in the left superior frontal region as low voltage fast readings propagating to the frontal pole as poly‐spikes. The patient received a left frontal lobectomy – outlined in red in (A). Image (B) depicts the inferior overview, (C) medial overview, and (D) anterior overview. The patient remains seizure‐free (ILAE 1) at 16 months postoperatively, with no functional deficits. (E) Intracranial EEG showing seizure onset in the left superior frontal region as low voltage fast readings propagating to the frontal pole as poly‐spikes

Resection was the most common treatment strategy (17/48, 35.4%). Topectomy was performed in 6/17 patients (35.3%), extended temporal lobectomy, and anteromedial temporal resection were performed in four patients each (23.5%). The remaining patients underwent lobar or sub‐lobar frontal or insular resection (Figure 2). The most common pathological findings in the resection group were: reactive gliosis (14/17, 82.4%), focal cortical dysplasia (FCD) type 2a (3/17, 17.6%) and FCD type 2b (1/17, 5.88%). The most common non‐resective treatment options were DBS (11), followed by RNS (10), VNS (5), corpus callosotomy (1), and LITT (1) (Figures S1 and S2). Significant associations with resective surgery included: icEEG monitoring (*P* = .003), ictal scalp EEG lateralization to the right hemisphere (OR 8.57, 95% CI: 2.18‐40.08), and icEEG lateralization to the right hemisphere (OR 11.2, 95% CI: 2.44‐67.1) (Table 2). All 17 patients who underwent resection received icEEG monitoring compared with 17/28 (60.7%) patients who underwent a non‐resective procedure (*P* = .003). A non‐lateralizing icEEG evaluation was highly associated with DBS treatment (6/7, 85.7%, patients with DBS vs 4/27, 14.8%, who received focal treatment, *P* < .001) as was a nonlocalizing icEEG evaluation (4/7, 57.1%, vs 2/27, 7.4%, *P* = .01). Preoperatively, the ictal scalp EEG was more commonly nonlocalizing in the DBS and VNS group (6/16, 37.5% vs 2/28, 7.1%, *P* = .019) and patients with these non‐focal treatments also had a preponderance to focal impaired awareness seizures (14/16, 87.5% vs 17/28, 60.7%). In the DBS cohort, 4/11 patients went directly to treatment without icEEG monitoring and these patients more commonly had secondarily generalized seizures (2/4 who received direct treatment vs 0/7 who received icEEG monitoring prior to DBS, *P* = .109) and nonlocalizing ictal scalp EEG (3/4 vs 1/7, *P* = .088). Two patients had complications following definitive surgical treatment (2/45, 4.44%): One had a wound infection of their DBS chest generator with removal and subsequent replacement, and the other suffered a common peroneal nerve palsy with subsequent resolution at 1 month.

**TABLE 2:** Significant predictors of undergoing resective surgery vs non‐resective surgery (neuromodulation, corpus callosotomy, LITT)

Variable	Resection	Non‐resective	*P* value	OR (95% CI)
Versive head turning semiology	10 (58.8%)	5 (18.5%)	.006	6.29 (1.68‐26.8)
Semiology lateralization (right)	7 (41.2%)	2 (7.1%)	.017	9.1 (1.84‐68.6)
Multifocal epilepsy risk factor	3 (17.6%)	14 (51.9%)	.030	0.119 (0.039‐0.779)
Ictal EEG lateralization (right)	10 (58.8%)	4 (14.3%)	.003	8.57 (2.18‐40.08)
PET (localized)	15 (88.2%)	15 (57.7%)	.045	5.5 (1.21‐39.63)
icEEG monitoring performed	17 (100.0%)	17 (60.7%)	.003	—^a^
icEEG monitoring on language side	8 (53.3%)	16 (94.1%)	.013	0.071 (0.003‐0.497)
icEEG lateralization (right)	12 (70.6%)	3 (17.6%)	.005	11.2 (2.44‐67.1)

Note

Risk factors for multifocal epilepsy included a history of viral encephalitis, traumatic brain injury, febrile seizures, prematurity or epileptic encephalopathy, or the presence of autism spectrum disorder, vascular dementia or febrile infection‐related epilepsy syndrome.

^a^ Severely biased effect size estimate since all resected patients underwent an intracranial study.

### 3.4 Surgical outcome

**FIGURE 3:**
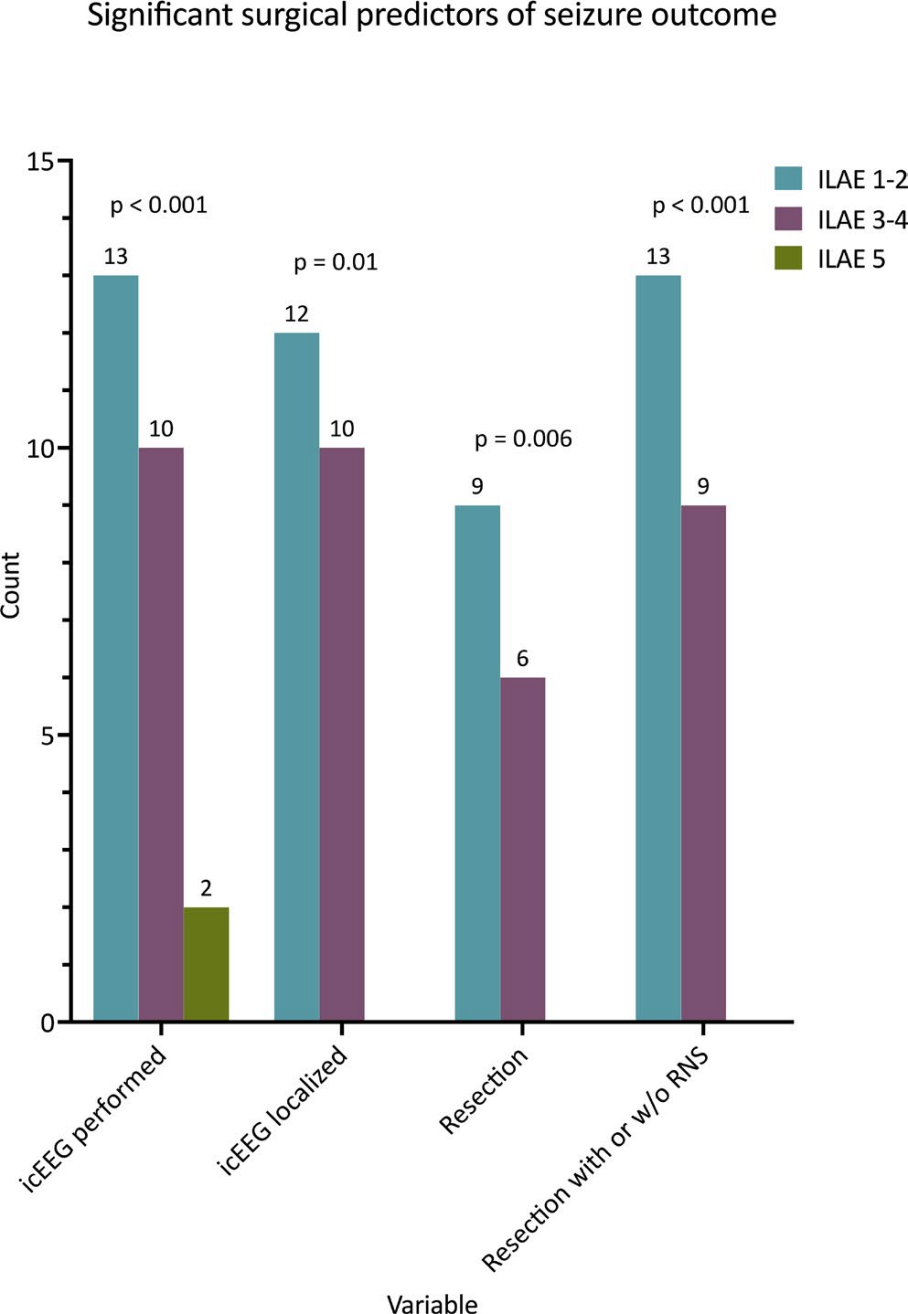
Significant predictors of good or excellent seizure outcome based on icEEG monitoring and the surgical intervention performed

**FIGURE 4:**
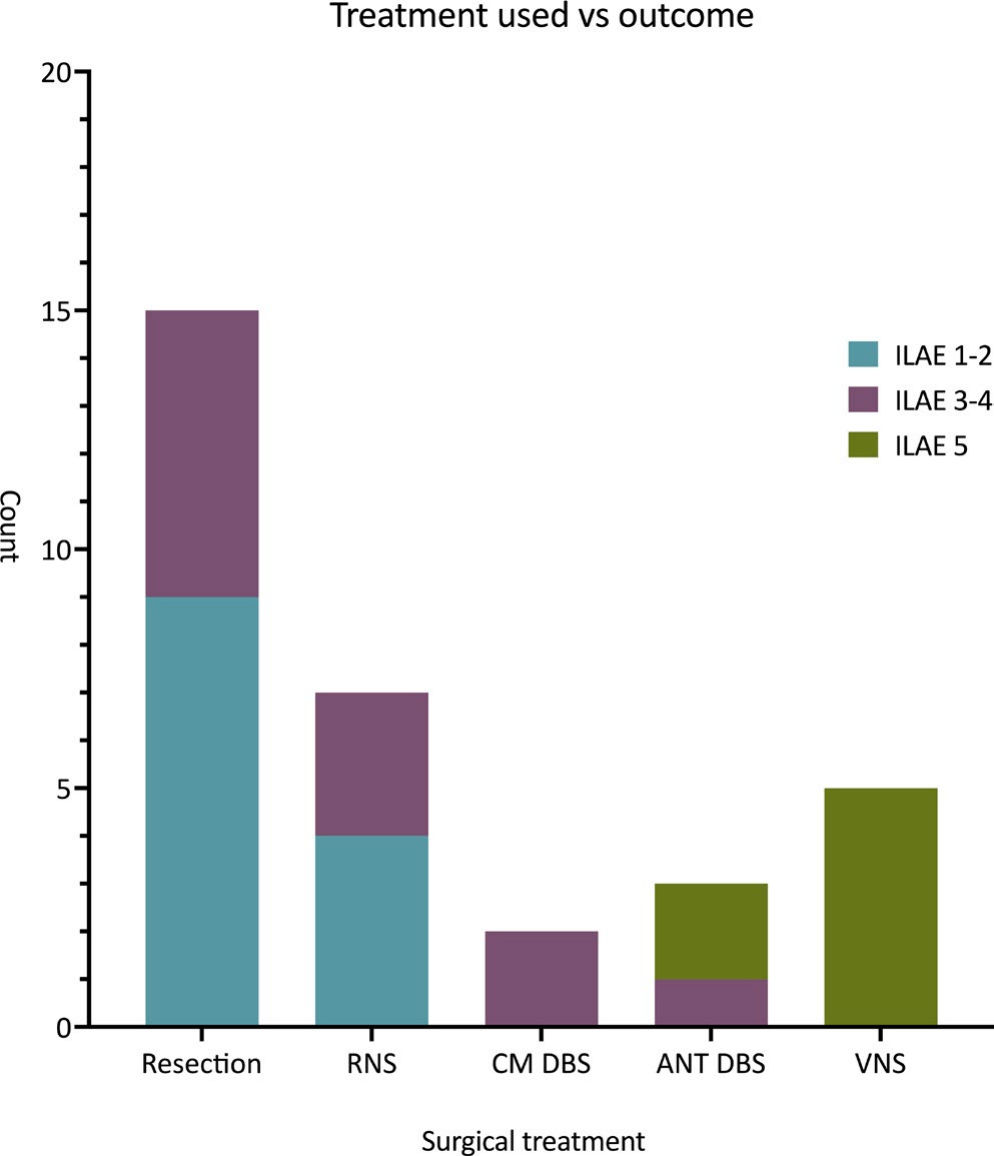
Comparison of ILAE outcomes by surgical intervention performed. The patient with LITT is not included in the figure due to the significant bias associated with having a single patient

Thirty‐three patients (68.8%) were included in the outcome analysis. Excellent outcome was achieved in 13 (39.4%) patients, good outcome in 12 (36.4%) and poor outcome in 8 (24.2%). Focal aware seizures were significantly associated with excellent outcomes (*P* = .047, Table S2). No other preoperative characteristics separated the groups, though a trend was observed toward better outcomes with a localizing ictal scalp EEG (*P* = .058). Most patients who achieved an excellent (ILAE 1‐2) or a good outcome (ILAE 3‐4) underwent icEEG monitoring (100% and 83.3%, respectively) compared with 25% who had icEEG monitoring with a subsequent poor outcome (*P* < .001). Patients with an excellent outcome were also more likely to have had resection or RNS treatment (69% and 31% respectively, *P* < .001, Figure 3). The treatments with the highest excellent outcome rates were resection (60%) and RNS (57.1%), and no patients in either group had a poor outcome. The worst performing group was VNS with a poor outcome in all five patients (Figure 4).

## 4 DISCUSSION

Our study showcases the array of contemporary surgical options available for medically intractable MRI‐negative epilepsy and their outcomes. While this new armamentarium expands treatment options, the plethora of resources poses a challenge: how does one choose between icEEG monitoring (which provides a chance for focal treatment) and directly pursuing neuromodulation? In our cohort, we performed icEEG monitoring in all patients with a negative MRI unless their preoperative evaluation strongly suggested a nonlocalizable epilepsy, which was usually indicated by the ictal scalp EEG. In these cases, we pursued non‐focal treatment, such as DBS or VNS. Aside from the ictal scalp EEG, there were few robust preoperative predictors of outcome. Thus, icEEG monitoring was the most reliable diagnostic investigation to guide treatment and achieve the best outcomes. Most patients who underwent icEEG monitoring were benefited due to seizure localization and subsequent resection and/or RNS, resulting in excellent outcomes in around 60% of cases. This is comparable with the outcomes reported in previous resection‐only series where 30%–60% seizure freedom has been demonstrated.^9^, ^10^ Even in patients with bilateral depth electrode‐only icEEG monitoring, which was used when the preoperative evaluation did not strongly lateralize, the majority underwent focal therapy with 7/11 having resection or RNS treatment.

In cases where resection carries a risk of postoperative functional deficit, RNS may be an excellent alternative treatment.^14^ In our study, 22% of patients underwent RNS alone and more than 50% had excellent outcomes, which is comparable to the seizure freedom rates reported in long‐term studies.^15^, ^16^ We performed icEEG monitoring, usually consisting of grid, strip and depth electrodes, prior to RNS treatment in all cases. This approach enabled precise localization of the seizure onset area and allowed for functional stimulation mapping, aiding the selection and placement of RNS in areas where language, motor, or sensory responses are found.^17^ In the medial temporal lobe, which comprised a large proportion of our cases, the effectiveness of RNS for treating seizures is independent of the presence or absence of a lesion whereas in the neocortex, it may be more effective in lesional cases.^14^, ^18^

Neuromodulatory treatment for nonlocalizable, MRI‐negative epilepsy in the United States was limited to VNS until 2018 when DBS was approved by the FDA. Several studies have shown the promise of DBS, with prospective studies indicating a 50% seizure reduction in 50%‐70% of patients at long‐term follow‐up.^19^, ^20^, ^21^, ^22^, ^23^ Similarly, 60% of our patients treated with DBS responded with greater than 50% seizure reduction – many of these had focal impaired awareness seizures which can be effectively treated with DBS.^20^, ^24^, ^25^ However, over 60% of our cohort that received DBS treatment underwent prior icEEG monitoring. Intracranial EEG monitoring carries risks, such as bleeding and infection and can cause significant postoperative discomfort.^26^, ^27^, ^28^ Analysis of our cohort revealed that a nonlocalizing ictal scalp EEG was associated with non‐focal therapy (including DBS) as the final treatment plan. This may assist in a priori identification of candidates who are suitable for direct DBS therapy. Since 2018, we have performed DBS in difficult to localize cases. In our cohort, two patients received VNS following the introduction of DBS. The decision to implant VNS was made on the basis of their comorbidities: one had vascular dementia, the other had a history of depression with multiple suicide attempts. VNS has been shown to be effective for the treatment of both depression and epilepsy,^29^, ^30^, ^31^ and it may improve cognitive function in neurodegenerative disorders.^32^, ^33^, ^34^ Future studies may investigate the decision‐making process regarding when DBS or VNS is indicated in MRI‐negative patients.

Two patients suffered complications from icEEG monitoring (2/37, 5.41%) and two patients had complications following definitive surgical treatment (2/45, 4.44%). No patient suffered permanent neurological injury or long‐term functional deficits. Prior studies have reported icEEG complication rates of around 10% which tends to decrease with time and experience,^28^, ^35^, ^36^ and complications from definitive treatment in around 5%‐15% of cases.^37^, ^38^ The relatively low rate of complications observed in this study may be attributable to the decades of experience with icEEG monitoring at our institution, and the relatively high rate of subsequent non‐resective procedures which carry a lower risk of complications. Patients with refractory epilepsy may benefit from surgery through reduction in seizures, improvement in neuropsychiatric symptoms, and quality of life outcomes among others, and these should be weighed against the risks of surgical failure and complications.^39^ If the patient may conceivably receive a focal therapy – and therefore has a good chance of seizure freedom – we would recommend icEEG monitoring for precise localization of the ictal onset.

Our study has several limitations including those inherent to its retrospective methodology such as selection bias and the challenge of collecting accurate outcomes data. Of the MRI‐negative patients presented at our multidisciplinary conference, 48 (27.4%) received a surgical intervention which is a similar proportion to previous reports.^40^ Patients are discussed at conference due to difficult to treat epilepsy and are considered for possible future surgical management or for continued medical therapy with a change to the therapeutic regimen. The majority of patients who did not receive a surgical intervention continued to receive medical therapy for their epilepsy based on a consensus decision. Our study might have suffered from selection bias since we would readily recommend icEEG monitoring in patients who are more likely to be localizable and therefore amenable to focal therapies, which confers improved outcome. The small cohort size for certain treatment modalities, such as LITT and VNS is also a major limitation and reduces our ability to generalize our findings for these forms of treatment. We reported outcomes at a minimum of 1 year as an indicator of long‐term outcomes. Our timeline is supported by a recent, large study showing no significant difference in outcomes at 1 year and 10 years postoperatively in MRI‐negative epilepsy patients treated with resection.^9^ However, neuromodulation outcomes may require longer follow‐up due to the continued improvement in seizure burden beyond the first year.^15^, ^16^, ^19^ Another limitation lies in the inclusion of treatment modalities with modest outcomes, such as VNS, which is often pursued when a patient is unlikely to receive focal treatment or is sometimes used as a salvage therapy following failed resection.^41^ This lack of control for treatment modality could have confounded and reduced our preoperative associations with outcome.

In conclusion, the decision of whether to perform icEEG monitoring or to directly pursue global neuromodulation (ie, DBS and VNS) in patients with MRI‐negative epilepsy presents a clinical quandary. Direct neuromodulation is an attractive option since some MRI‐negative patients are not eligible for resection or RNS following icEEG monitoring. Further, direct neuromodulation may be associated with increased patient comfort and a risk reduction by not undergoing icEEG monitoring. Our study demonstrates that an icEEG evaluation followed by resection and/or RNS may offer a better chance of seizure freedom in patients with intractable MRI‐negative epilepsy and that, if carefully selected, a good proportion of patients may benefit from an icEEG study. Complete seizure control provides the best quality of life improvement for the patient and should therefore be the goal of treatment where possible.^42^ In a proportion of cases, it is difficult to tailor treatment based on the preoperative evaluation alone, thus the icEEG study may offer a useful tool for surgical decision‐making and for optimizing the treatment of each patient. VNS or DBS may be pursued directly in cases where the seizure semiology and preoperative evaluation are highly indicative of multifocal epilepsy; however, more data are needed to support this approach.

## CONFLICT OF INTEREST

ECD reports grant funding from the NIH (KL2 TR001862, NeuroNEXT U24NS107136, ARDC P30AG066508). The remaining authors have nothing to report. We confirm that we have read the Journal's position on issues involved in ethical publication and affirm that this report is consistent with those guidelines.

## AUTHOR CONTRIBUTIONS

HM, MM, MRSS, DDS and ECD conceived the idea; HM, MM and MRSS acquired the data; HM, MM and MRSS carried out the statistical analyses and provided the figures/tables; HM, MM, MRSS, DDS and ECD interpreted the data; HM, MM, MRSS, LL, NAM, PF, DDS and ECD wrote the manuscript. All authors read and approved the final submitted manuscript. HM had full access to all the data in the study and takes responsibility for the integrity of the data and the accuracy of the data analysis.

## Supporting information

Figure S1

Figure S2

Table S1

Table S2
