# Supplementary figures and images for: Optimizing the surgical management of MRI‐negative epilepsy in the neuromodulation era
Source: Epilepsia Open. 2022 Feb 1;7(1):151–9. doi: 10.1002/epi4.12578 (PMC8886105; doi:10.1002/epi4.12578)

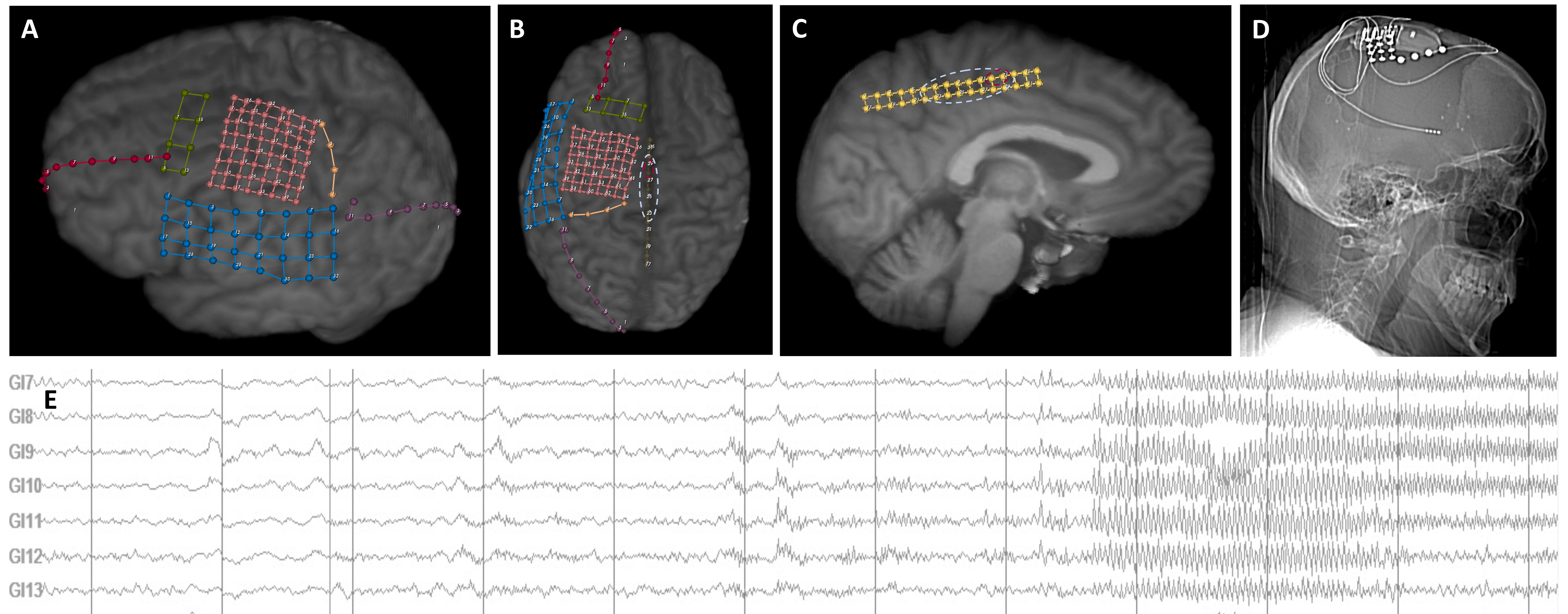

Supplement: Supplementary file 1 — Figure S1 [file EPI4-7-151-s001.tif]

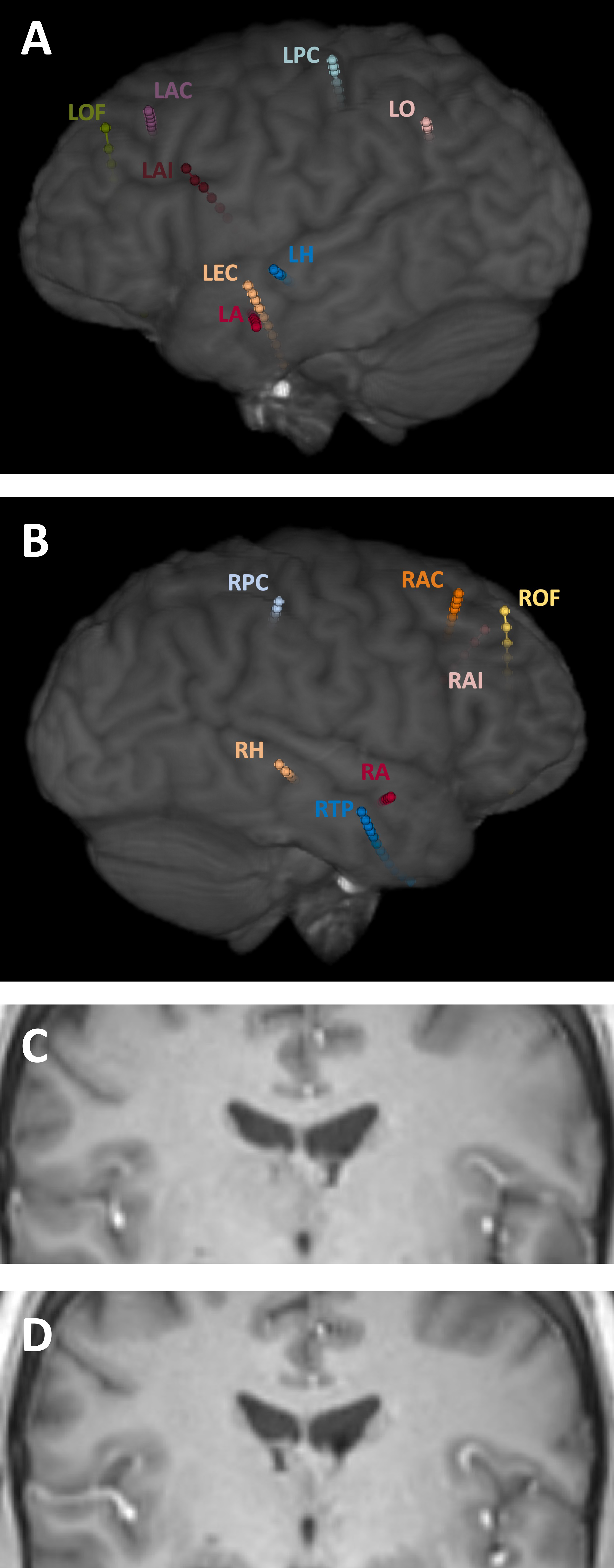

Supplement: Supplementary file 2 — Figure S2 [file EPI4-7-151-s002.tif]
