# Supplementary material for: Optimizing the surgical management of MRI‐negative epilepsy in the neuromodulation era
Source: Epilepsia Open. 2022 Feb 1;7(1):151–9. doi: 10.1002/epi4.12578 (PMC8886105; doi:10.1002/epi4.12578)
Supplement: Supplementary file 3 — Table S1 [file EPI4-7-151-s003.docx]

**Table S1.** Descriptive statistics of the cohort.

| Variable | Total (n = 48) |
| --- | --- |
| Age (years) | 32.8 (± 12.7) |
| Sex (female) | 25 (52.1%) |
| Handedness (right) | 43 (89.6%) |
| Ethnicity (Caucasian) | 35 (72.9%) |
| Psychiatric comorbidities | 19 (39.6%) |
| Age at epilepsy onset (years) | 15 (± 12.6) |
| Duration of epilepsy (years) | 18.25 (± 9.69) |
| Number of seizures/month | 40 (± 66.9) |
| Habitual seizure characteristics  Focal aware seizures  Focal impaired aware seizures  Generalized seizures | 22 (45.8%)  33 (68.8%)  10 (20.8%) |
| Semiology localization  Temporal  Fronto-temporal  Frontal  Temporo-occipital  Occipital  Fronto-parietal  Multifocal | 17 (35.4%)  8 (16.7%)  7 (14.6%)  2 (4.17%)  1 (2.08%)  1 (2.08%)  12 (25%) |
| Ictal scalp EEG localization  Temporal  Fronto-temporal  Frontal  Occipital  Parietal  Multifocal | 22 (45.8%)  4 (8.33%)  10 (20.8%)  1 (2.08%)  3 (6.25%)  8 (16.7%) |
| Underwent intracranial study | 37 (77.1%) |
| Intracranial localization  Mesial temporal  Temporal neocortical  Frontal  Fronto-temporal  Occipital  Parietal  Fronto-parietal  Insula  Temporo-parietal  Incomplete investigation  Multifocal | 10 (27%)  6 (16.2%)  4 (10.8%)  3 (8.1%)  2 (5.41%)  1 (2.7%)  1 (2.7%)  1 (2.7%)  1 (2.7%)  1 (2.7%)  7 (18.9%) |
| Underwent resection | 17 (35.4%) |
| Underwent non-resective treatment | 28 (58.3%) |
| Not treated | 3 (6.25%) |
