# Supplementary material for: Optimizing the surgical management of MRI‐negative epilepsy in the neuromodulation era
Source: Epilepsia Open. 2022 Feb 1;7(1):151–9. doi: 10.1002/epi4.12578 (PMC8886105; doi:10.1002/epi4.12578)
Supplement: Supplementary file 4 — Table S2 [file EPI4-7-151-s004.docx]

**Table S2.** Results of the analysis of presurgical associations with seizure outcome at 1-year following treatment. ^†^ P ≤ 0.05

| **Variable** | **ILAE 1-2** | **ILAE 3-4** | **ILAE 5** | **P value** |
| --- | --- | --- | --- | --- |
| FA seizures^†^ | 9 (69.2%) | 3 (25%) | 2 (25.0%) | 0.047 |
| Neuropsychology lateralization (lang dominant) | 4 (57.1%) | 5 (100%) | 1 (100%) | 0.388 |
| Neuropsychology lateralization (lateralized) | 8 (61.5%) | 5 (50%) | 1 (16.7%) | 0.221 |
| Neuropsychology localization (localized) | 10 (83.3%) | 4 (44.4%) | 3 (50.0%) | 0.175 |
| Interictal EEG lateralization (right) | 4 (30.8%) | 3 (25%) | 0 (0%) | 0.237 |
| Interictal EEG localization (localized) | 9 (69.2%) | 10 (83.3%) | 6 (75.0%) | 0.873 |
| Ictal EEG lateralization (right) | 7 (53.8%) | 3 (25%) | 1 (12.5%) | 0.129 |
| Ictal EEG localization (localized) | 13 (100%) | 9 (75%) | 5 (62.5%) | 0.058 |
| PET lateralization (right) | 6 (46.2%) | 5 (45.5%) | 1 (14.3%) | 0.367 |
| PET localization (localized) | 10 (76.9%) | 9 (81.8%) | 3 (42.9%) | 0.238 |
| SPECT lateralization (right) | 1 (16.7%) | 1 (25%) | 0 (0%) | 0.714 |
| SPECT localization (localized) | 3 (60%) | 3 (75%) | 3 (60.0%) | 1 |
| Ictal scalp EEG-PET concordance | 6 (46.2%) | 2 (18.2%) | 1 (14.3%) | 0.271 |
| Ictal scalp EEG-SPECT concordance | 0 (0%) | 0 (0%) | 1 (20%) | 0.6 |
| Scalp EEG concordance (>2 modalities) | 2 (15.4%) | 3 (25%) | 2 (25%) | 0.758 |
